# Activity of the mouse Notch ligand DLL1 is sensitive to C-terminal tagging in vivo

**DOI:** 10.1186/s13104-021-05785-4

**Published:** 2021-09-28

**Authors:** Karin Schuster-Gossler, Karsten Boldt, Dorothee Bornhorst, Patricia Delany-Heiken, Marius Ueffing, Achim Gossler

**Affiliations:** 1 Institute for Molecular Biology, OE5250, Hannover Medical School, Carl-Neuberg-Str. 1, 30625 Hannover, Germany; 2 Institute of Ophthalmic Research, Center for Ophthalmology, University of Tübingen, Elfriede-Aulhorn-Strasse 7, 72076 Tübingen, Germany; 3 Institute of Biochemistry and Biology, Potsdam University, 14476 Potsdam, Germany; 4 Present Address: Stem Cell Program and Division of Hematology/Oncology, Boston Children’s Hospital, Boston, USA; 5 Present Address: Department of Stem Cell and Regenerative Biology, Harvard University, Cambridge, USA

**Keywords:** Mouse DLL1, DLL1 C-terminal tagging, AcGFP, StrepFLAG, DLL1 hypomorph

## Abstract

**Objective:**

The mammalian Notch ligand DLL1 has essential functions during development. To visualise DLL1 in tissues, for sorting and enrichment of DLL1-expressing cells, and to efficiently purify DLL1 protein complexes we tagged DLL1 in mice with AcGFPHA or Strep/FLAG.

**Results:**

We generated constructs to express DLL1 that carried C-terminal in-frame an AcGFPHA tag flanked by loxP sites followed by a Strep/FLAG (SF) tag out of frame. Cre-mediated recombination replaced AcGFP-HA by SF. The AcGFPHAstopSF cassette was added to DLL1 for tests in cultured cells and introduced into endogenous DLL1 in mice by homologous recombination. Tagged DLL1 protein was detected by antibodies against GFP and HA or Flag, respectively, both in CHO cells and embryo lysates. In CHO cells the AcGFP fluorophore fused to DLL1 was functional. In vivo AcGFP expression was below the level of detection by direct fluorescence. However, the SF tag allowed us to specifically purify DLL1 complexes from embryo lysates. Homozygous mice expressing AcGFPHA or SF-tagged DLL1 revealed a vertebral column phenotype reminiscent of disturbances in AP polarity during somitogenesis, a process most sensitive to reduced DLL1 function. Thus, even small C-terminal tags can impinge on sensitive developmental processes requiring DLL1 activity.

**Supplementary Information:**

The online version contains supplementary material available at 10.1186/s13104-021-05785-4.

## Introduction

The *Dll1* gene encodes a mammalian Notch ligand, is expressed in complex patterns in numerous cell types and tissues [1–7] and critical for example during somite patterning and myogenic differentiation [8–10], during vascular development [11–13] and for establishment of left–right asymmetry [14, 15], differentiation of pancreatic [16], neuronal [17], epidermal [18], marginal zone B [19] and intestinal stem cells [20].

To analyse the dynamics of DLL1 protein expression by live imaging endogenous DLL1 was previously C-terminally tagged with three luciferase proteins [21]. Fusion with red luciferase was fully functional, whereas a DLL1 firefly luciferase fusion was slightly hypomorphic and a DLL1 emerald luciferase fusion was non-functional [21]. Thus, C-terminal tagging of DLL1 without compromising DLL1 function is -in principle- possible.

Here, we tagged endogenous DLL1 at its C-terminus by homologous recombination. We chose monomeric AcGFP [22] as a means to detect and isolate DLL1 expressing cells and a SF tag to affinity purify DLL1 complexes for mass spectrometric analyses.

## Main text

### Methods

Here, methods are briefly summarised. For a detailed description of all materials, primers, antibodies and methods please see Additional file 1.

#### Mice

Mice expressing tagged DLL1 were generated in this study, ZP3::Cre [23] and FLPe mice [24] were described previously.

#### Cells

CHO and ES cells were used in this study.

#### Constructs

A gene fragment encoding AcGFP [22] fused with an HA Tag followed by a stop codon, flanked by loxP sites followed by a SF tag [25] and a stop codon was synthesized and used to generate expression and targeting vectors by standard cloning procedures.

#### Cell surface biotinylation

Cell surface presentation of DLL1variants was analysed in CHO cells.

#### Gene targeting, and generation of mice

ES cells were electroporated, clones screened by PCR and validated by Southern blot hybridisations and used to generate *Dll1*^*AcGFPHAstopSF*^ mice.

#### Skeletal preparations

Skeletal preparations were stained by Alcian blue and Alizarin red.

#### Confocal imaging

Images were acquired using a Leica SP8 confocal laser microscope using the Las X Software (Leica) and processed with Adobe Photoshop CS5.

#### Immunoprecipitation (IP)

IPs of DLL1 variants were done with anti-GFP or anti-HA or anti-Flag antibodies and Sepharose G beads.

#### Affinity purification of DLL1 complexes and mass spectrometry

DLL1 complexes were affinity purified from E10.5 embryos and analysed by LC–MS/MS as described [26].

### Results

**Fig. 1:**
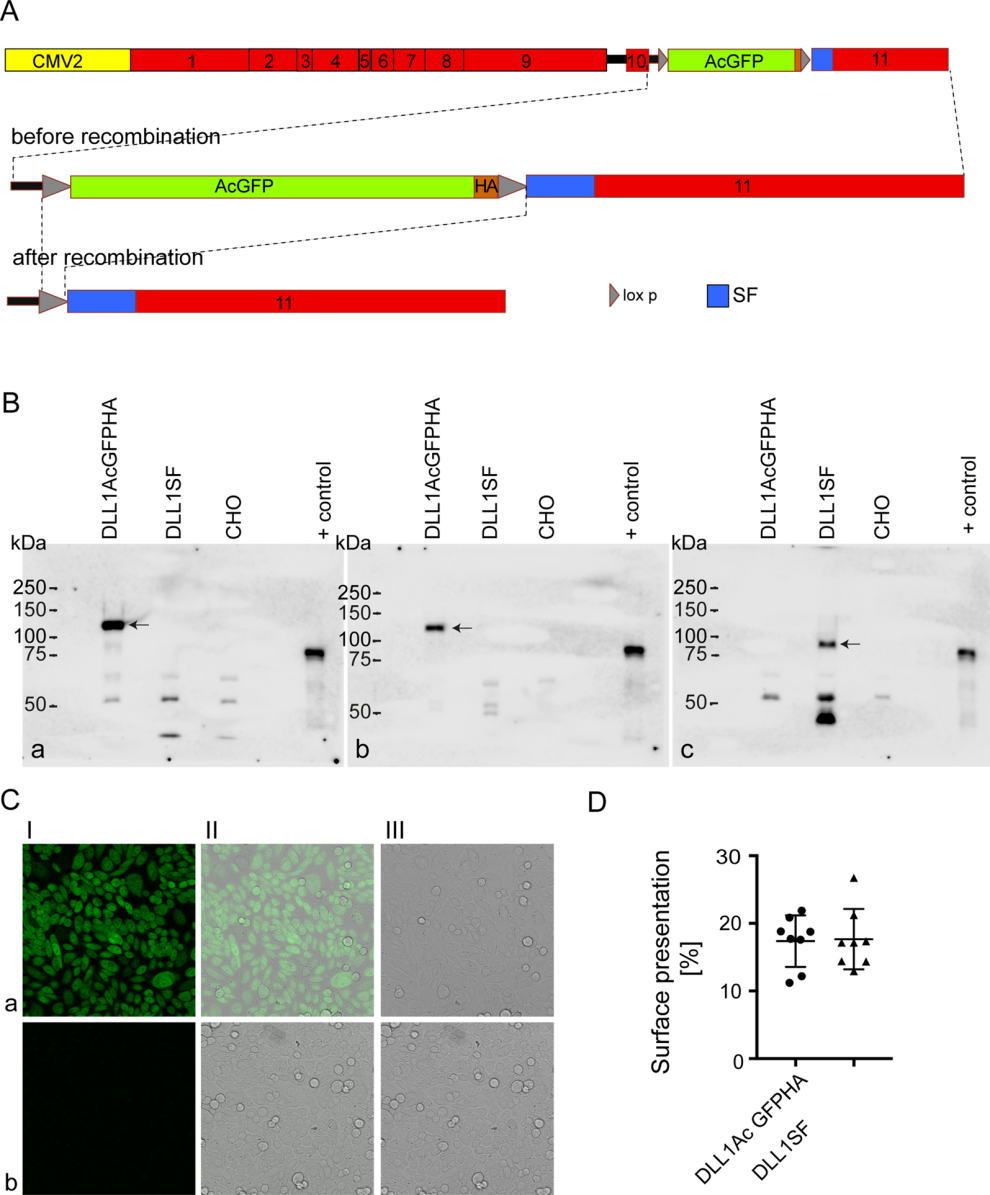
Analysis of DLL1AcGFPHA and DLL1SF proteins in CHO cells. **A** Scheme of constructs used to express DLL1AcGFPHA or DLL1SF in CHO cells under control of the CMV2 promoter. Full length construct (top), 3’ region before (middle), and after (bottom) Cre-mediated recombination. **B** Expression of DLL1 variants in CHO cells carrying DLL1AcGFPHA or DLL1SF. DLL1 variants were immunoprecipitated with anti HA (**a**) anti-GFP (**b**) or anti-Flag (**c**) antibodies and detected in Western blots using anti-DLL1 antibody 1F9. + control: Lysate of CHO cells stably overexpressing DLL1. Arrows point to tagged DLL1 proteins. Photographs of the Western blot membranes are shown in Additional file 7: Figure S3. **C** AcGFP fluorescence in DLL1AcGFPHA expressing CHO cells (row a) in comparison to wild-type CHO cells (row b); Column I: fluorescence, II: overlay, III: bright field. **D** Surface presentation of DLL1AcGFPHA (dots) and DLL1SF (triangles) in CHO detected in cell surface biotinylation assays

Prior to tagging endogenous DLL1 we tested our strategy and functionality of the tags in cultured cells. A *Dll1* expression construct was cloned into pCMV2 using the *Dll1* cDNA with the AcGFPHAstopSF cassette fused to the C-terminus of DLL1 (Fig. 1A), a modification identical to the one planned to tag endogenous DLL1. CHO cells were generated with stably integrated pCMV2Dll1AcGFPHAstopSF or pCMV2Dll1SF (the latter obtained by recombination of the Dll1AcGFPHAstopSF plasmid in Cre-expressing bacteria). Due to high background observed with the HA antibody in Western blots in CHO cell lysates expression of tagged DLL1 was analysed by immunoprecipitation with anti-GFP, -HA and -Flag antibodies including CHO wild type cells as controls. Precipitated proteins were detected by western blot analyses using the DLL1-specific monoclonal antibody 1F9, lysates of DLL1Flag overexpressing CHO cells [27] served as positive controls. DLL1AcGFPHA was detected in CHO cells carrying pCMV2Dll1AcGFPHAstopSF after IP with anti-GFP and HA antibodies (Fig. 1B a, b) but as expected not after IP with the anti-Flag antibody (Fig. 1B c). CHO cells carrying pCMV2Dll1SF showed no detectable signal with 1F9 after IP with anti-GFP or HA antibodies (Fig. 1B a, b) but showed expression of DLL1SF after IP with the anti-flag antibody (Fig. 1B c). Thus, as planned, a differently tagged DLL1 variant was obtained after Cre-mediated recombination of pCMV2Dll1AcGFPHAstopSF replacing AcGFPHA with the SF tag. The functionality of AcGFP was confirmed by confocal fluorescence microscopy of DLL1AcGFPHA expressing CHO cells (Fig. 1C). Surface presentation of the tagged DLL1 proteins was investigated by biotinylation of CHO cells expressing DLL1AcGFPH or DLL1SF. Both variants were detected at the cell surface at similar levels (Fig. 1D, Additional file 3: Figure S2A, B, Additional file 4: Table S1 and Additional file 5: Table S2). In addition to the tagged DLL1 proteins migrating at the expected molecular weights a shorter DLL1 protein was detected in these assays with both variants (Additional file 3: Figure S2A, B). Cell surface biotinylation followed by Avidin pull down or IP with anti-GFP or anti-Flag antibodies showed that the faster migrating DLL1 proteins lacked the C-terminal tags (Additional file 3: Figure S2C) suggesting that the tags were removed from DLL1 by proteolytical cleavage. Since “cleaved” DLL1 was less abundant in cell lysates than in the affinity-purified fraction (Additional file 4: Table S1 and Additional file 5: Table S2) removal of the tag might at least in part occur during the purification despite the presence of protease inhibitors.

**Fig. 2:**
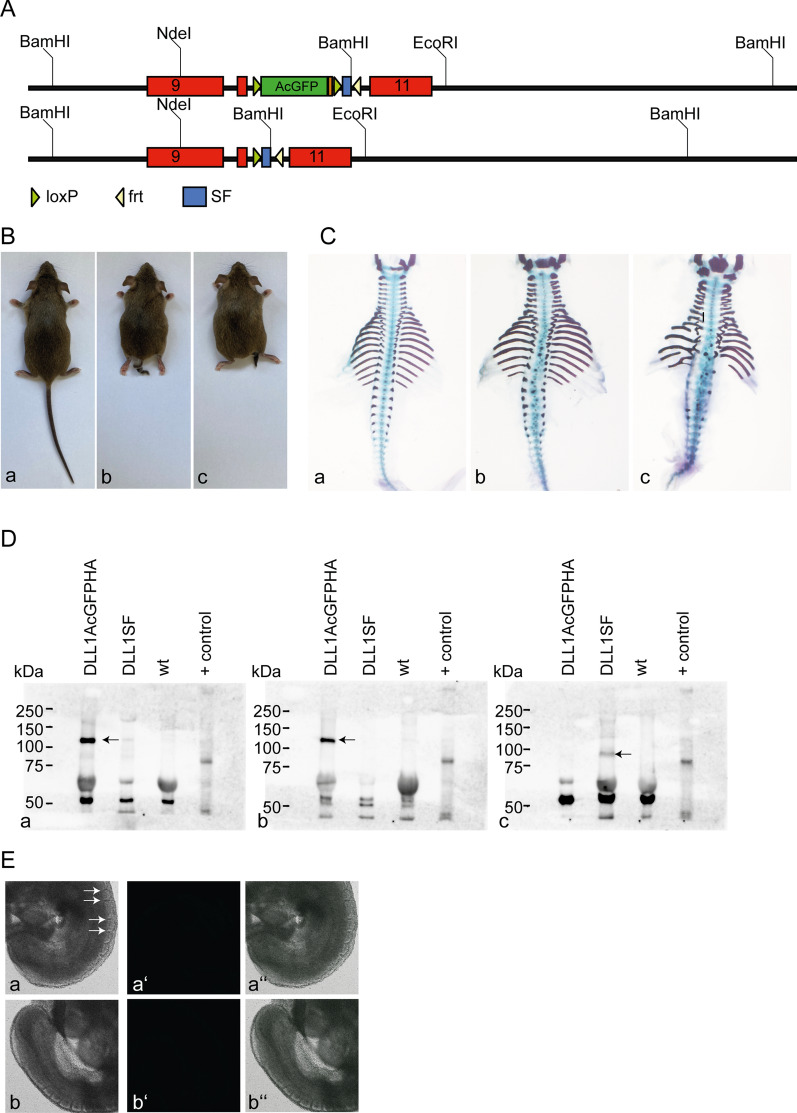
Analysis of *Dll1*^*AcGFPstopSF*^ and *Dll1*^*SF*^ mice. **A** Scheme of the modified 3’ region before (top) and after (bottom) Cre-mediated recombination. **B** Phenotype of wt (**a**), homozygous adult *Dll1*^*AcGFPHAstopSF*^ (**b**) and *Dll1*^*SF*^ (**c**) mice. **C** Skeletal preparations of wt (**a**), homozygous *Dll1*^*GFPHAstopSF*^ (**b**) and *Dll1*^*SF*^ d15.5 fetuses. **D** Detection of DLL1 variants in homozygous *Dll1*^*AcGFPHAstopSF*^, *Dll1*^*SF*^ and wild type d10.5 embryos. DLL1 variants were immunoprecipitated with anti-HA (**a**), anti-GFP (**b**) or anti-Flag (**c**) antibodies and detected in Western blots using anti-DLL1antibody 1F9. + control: Lysate of DLL1 overexpressing ES cells. Arrows point to tagged DLL1 proteins. Photographs of the Western blot membranes are shown in Additional file 8: Figure S4. **E** GFP fluorescence in homozygous d9,5 *Dll1*^*AcGFPHAstopSF*^ (**a**–**a**’’) and wild type (**b**–**b**’’) embryos; (**a**, **b**) bright field, (**a**’, **b**’) fluorescence, (**a**’’, **b**’’) overlay. Arrows in (**a**) point to irregular somites

The C-terminal modifications were introduced into endogenous DLL1 by homologous recombination in ES cells. To increase the targeting frequency at the *Dll1* locus, which in our hands tended to be low, we employed the CRISPR/Cas system. Targeting events were first detected by PCR and validated by Southern blot analyses, which revealed a surprising high number of off-target events and multiple integrations (Additional file 2: Figure S1B). One correctly targeted ES cell clone transmitted the planned alteration (*Dll1*^*AcGFPHAstopSF*^) through the germ line. *Dll1*^*AcGFPHAstopSF*^ was recombined in the female germ line of ZP3:Cre mice to obtain the *Dll1*^*SF*^ allele. Heterozygous mice carrying either allele (*Dll1*^*AcGFPHAstopSF*^ n = 18 and *Dll1*^*SF*^ n = 18) were phenotypically normal. Homozygous mice of both alleles and sexes were viable. Adult mice (*Dll1*^*AcGFPHAstopSF*^ n = 24 and *Dll1*^*SF*^ n = 46) showed short and kinky tails suggesting vertebral column defects (Fig. 2B b,c). In addition, 60% (15/25) of test-mated homozygous males carrying the *Dll1*^*SF*^ allele were infertile. Skeletal preparations of E15.5 embryos (*Dll1*^*AcGFPHAstopSF*^ n = 10 and *Dll1*^*SF*^ n = 8) revealed misshaped vertebral bodies and ribs indicative of somite patterning defects, which appeared to be more severe in the *Dll1*^*SF*^ allele (Fig. 2C b,c). Expression of tagged DLL1 was analysed by immunoprecipitations from homozygous d10.5 embryo lysates followed by detection with anti-DLL1 1F9. In *Dll1*^*AcGFPHAstopSF*^ embryos DLL1 was detected after IP with anti-GFP or -HA antibodies and not after IP with anti-Flag (Fig. 2D a,b). In *Dll1*^*SF*^ embryos DLL1 was detected after IP with anti-Flag and not after IP with anti-GFP or -HA (Fig. 2D c). These findings confirmed that the *Dll1*^*AcGFPHAstopSF*^ allele recombined in mice and the anticipated tagged DLL1 proteins were generated. In contrast to DLL1AcGFPHA overexpressing CHO cells no fluorescence was detected in homozygous d9,5 *Dll1*^*AcGFPHAstopSF*^ embryos (n = 6; Fig. 2E a). This suggests that DLL1 expression levels in the transgenic CHO cells were significantly higher than from the endogenous locus and low endogenous DLL1 levels prevented detection of AcGFP fluorescence.

To analyse whether the SF tag allows one to purify sufficient DLL1 complexes for mass spectrometry we performed a pilot study using E10.5 embryo lysates and a one-step purification with anti-Flag affinity beads. Affinity complexes from wild type (control) and homozygous *Dll1*^*SF*^ embryos were purified in 6 independent experiments and analysed by mass spectrometry. DLL1 as well as 61 other proteins were detected specifically in the material purified from *Dll1*^*SF*^ embryos (Additional file 6: Table S3) demonstrating that Flag-tagged DLL1 was specifically purified in sufficient amount from transgenic mouse tissues for analysis by mass spectrometry. The full data set has been submitted to the PRIDE database (accession number PXD024680).

### Discussion

We have modified endogenous mouse DLL1 by homologous recombination in one step to tag DLL1 for observation in living cells and tissues, sorting of DLL1 expressing cells, or affinity purification to identify DLL1 protein complexes. The employed tags were functional in CHO cells but impinged on DLL1 function in vivo such that somite patterning, the process most sensitive to reduced DLL1 function [28] was affected. In addition, endogenous DLL1 tagged by AcGFP was not detected by fluorescence.

Previously DLL1 was C-terminally fused with three different luciferase proteins. Fusion with red luciferase was fully functional, whereas a DLL1 firefly luciferase fusion was hypomorphic and DLL1 fused to emerald luciferase was non-functional [21]. Our C-terminal fusions behave as hypomorphic alleles similar to but more severe than firefly luciferase.

Based on the published data [21] and our results (this paper) 4 out of 5 C-terminal tags affected DLL1 function to varying degrees, although in principle C-terminal tagging is possible without impinging on DLL1 activity. Reduction of DLL1 activity does not appear to depend on the length of the tag because the long red luciferase had no effect whereas the short SF tag (plus the peptide encoded by the *loxP* site) affected DLL1 function more strongly than our longer AcGFPHA tag. Removal of a 16 bp fragment in the 3´UTR might affect RNA stability and could be responsible for reduced DLL1 activity in our transgenic mice. However, mice tagged with firefly or emerald luciferase had a complete 3´ UTR [21] and also showed hypomorphic *Dll1* phenotypes. AcGFP was described as a monomeric protein [22, 29]. Thus, non-physiological clustering of DLL1AcGFPHA is an unlikely reason for reduced DLL1 function, although abnormal clustering and trafficking of C-terminally tagged DLL1 cannot be excluded. DLL1 carries a PDZ binding domain at its C-terminus which interacts with Acvrinp1, a MAGUK family member [30], and ARIP2, which has been implicated in stabilizing DLL1 and DLL4 [31]. A free C-terminus is important for interactions with PDZ proteins in many cases [reviewed in 32, 33]. C-terminal extension of DLL1 with tags might interfere with such interactions but this seems unlikely to play a major role as the C-terminal extension by red luciferase did not affect DLL1 function. Sequences of the C-terminal fusion appear to be an important factor and might affect protein stability or trafficking or processing in vivo and thereby impinge on DLL1 protein function.

AcGFP fused to DLL1 in DLL1AcGFPHA over expressing CHO cells was detected by direct fluorescence indicating that AcGFP in the context of the fusion protein is functional. However, in homozygous mouse embryos we did not detect AcGFP fluorescence in any tissue. A plausible explanation could be that expression levels in CHO cells were much higher than low levels of endogenous DLL1. Thus, absence of detectable fluorescence likely reflect DLL1AcGFPHA levels that are below the limit of detection of our set up. Whether fluorescing proteins with other activation and excitation properties and a better quantum yield (for example ZsGreen1 [29]) are sufficient to detect expression of a DLL1 fusion protein in vivo remains to be addressed.

Whereas DLL1AcGFPHA in our mice turned out to be insufficient for direct DLL1 detection by fluorescence our pilot study using the *Dll1*^*SF*^ allele demonstrated its usefulness for the purification of DLL1-containing protein complexes (Additional file 6: Table S3) from endogenous sources. Components of the secretory pathway and vesicle transport were identified, which can be expected for DLL1, a transmembrane protein that undergoes endocytotic processing [34]. Additionally, enzymes involved in ubiquitination copurified with tagged DLL1. Since DLL1 is modified by ubiquitin [34] also these potential interaction partners support that specific DLL1 protein complexes were affinity-purified. GO term analysis [35, 36] showed a surprising enrichment of other identified proteins implicated in metabolic processes, nucleotide-binding and catalytic activity. As far as we know, these proteins have not been implicated in or related to DLL1 function as yet and their significance for DLL1 activity will require further analyses. Given that our tag impinged on DLL1 function the tag might also prevent the isolation of a subset of DLL1 complexes.

In conclusion, DLL1 activity appears to be highly sensitive to sequences added to the C-terminus. Which sequences are tolerated by DLL1 are currently not predictable and might only be determined empirically by comprehensive studies in vitro and in vivo.

## Limitations

Whether cleavage of the C-terminal tag occurs in vivo and affects detection and function of tagged DLL1 is unclear, as is a potential effect of the unphysiological C-terminal fragments. Cleavage might remove the C-terminal PDZ domain and thereby affect protein interactions. Although enzymes involved in ubiquitination copurified with tagged DLL1 ubiquitination of the tagged versions might differ from wt DLL1 and contribute to reduced DLL1 function. Likewise, a potential effect of the peptide encoded by the loxP sequence cannot be ruled out.

## Supplementary Information

**Additional file 1: Text S1.** Detailed description of materials, primers, antibodies and methods.

**Additional file 2: Figure S1.** Targeting of *Dll1.* **(A)** Targeting scheme (for details see Material and Methods). **(B)** Southern blots of BamHI-digested genomic ES cell DNA with radioactively labelled probes from the 5’ flank (a), the 3’flank (b), and puro (c). Left lanes at each panel show ethidium bromide-stained lanes from agarose gels. Correctly targeted clones are indicated at the top. wt = wild type ES DNA.

**Additional file 3: Figure S2.** Surface biotinylation of tagged DLL1 proteins. **(A)** Western blots of cell lysates (input) and biotinylated proteins purified by Avidin beads (Avpd) from CHO cells expressing DLL1AcGFPHA. (a) Photograph of bound antibodies detected by chemoluminescence, (b) overlay of bright field and chemoluminescence photographs of Western blot membranes. Two aliquots from each of the 8 samples analysed per cell line (x.1 and x.2) were quantified relating the input to the Avpd band. Dotted lines indicate where membranes were cut. Primary antibodies used are indicated to the right. **(B)** Western blots of cell lysates (input) and biotinylated proteins purified by Avidin beads (Avpd) from CHO cells expressing DLL1SF. (a) Photograph of bound antibodies detected by chemoluminescence, (b) overlay of bright field and chemoluminescence photographs of Western blot membranes. Two aliquots from each of the 8 samples analyzed per cell line (x.1 and x.2) were quantified relating the input to the Avpd band. Dotted lines indicate where membranes were cut. Primary antibodies used are indicated to the right. **(C)** Western blots of cell lysates (input) and biotinylated proteins purified by Avidin beads (Avpd) or immunoprecipitated with anti-GFP (IP GFP) or anti-Flag (IP Flag) antibodies from CHO cells expressing DLL1AcGFPHA (left) or DLL1SF (right). (a) Photograph of bound antibodies detected by chemoluminescence**, (b)** overlay of bright field and chemoluminescence photographs of Western blot membranes. Dotted lines indicate where membranes were cut. Primary antibodies used are indicated to the right. DLL1Flag: lysate of CHO cells expressing flag-tagged DLL1 serving as positive control. Arrows point to biotinylated DLL1 purified by Avidin beads that is not immunoprecipitated by anti-GFP or anti-Flag antibodies. Asterisks indicate Ig heavy chains of primary antibodies used for immunoprecipitations detected by the secondary antibodies.

**Additional file 4: Table S1.** Quantification of DLL1AcGFPHA cell surface presentation. The determined values of inputs (full length and cleaved product) were multiplied with the factor 50 and the Avidin pull downs (full length and cleaved product) with the factor 2,2 to calculate the total amount of detected DLL1 in the lysate and IP in each sample. Each sample was analysed twice (#x.1 and #x.2).

**Additional file 5: Table S2.** Quantification of DLL1SF cell surface presentation. The determined values of inputs (full length and cleaved product) were multiplied with the factor 50 and the Avidin pull downs (full length and cleaved product) with the factor 2,2 to calculate the total amount of detected DLL1 in the lysate and IP in each sample. Each sample was analysed twice (#x.1 and #x.2).

**Additional file 6: Table S3.** Proteins detected in DLL1 complexes. Listed are significantly detected proteins. The full mass spectrometry data are available in the PRIDE database under Accession number PXD024680.

**Additional file 7: Figure S3.** Overlay of bright field and chemoluminescence photographs of the Western blot membranes used for Fig. 1Ba-c. a-c correspond to a-c in Fig. 1B.

**Additional file 8: Figure S4.** Overlay of bright field and chemoluminescence photographs of the Western blot membranes used for Fig. 2D a-c. a-c correspond to a-c in Fig. 2D.

## Data Availability

All data supporting the results of this article are included in this article and its additional files, the full mass spectrometry data are available openly in the PRIDE database under Accession number PXD024680 following publication.

## Abbreviations

3´UTR: 3’ Untranslated region
AcGFP: Aequorea coerulescens green fluorescent protein
AP: Anterior–posterior
Avpd: Avidin pull-down
CHO: Chinese Hamster Ovary
DLL1: Delta-like 1
ES: Embryonic stem
GO: Gene ontology
HA: Hemagglutinin
IP: Immunoprecipitation
LC–MS/MS: Liquid-chromatography-mass spectrometry/mass spectrometry
pCMV2: Cloning vector containing cytomegalovirus promoter
PDZ: Domain present in PSD-95, Dlg and ZO1/2
SF tag: Strep/FLAG tandem affinity purification tag
wt: Wild type
