# Supplementary material for: Activity of the mouse Notch ligand DLL1 is sensitive to C-terminal tagging in vivo
Source: BMC Res Notes. 2021 Sep 28;14:383. doi: 10.1186/s13104-021-05785-4 (PMC8477538; doi:10.1186/s13104-021-05785-4)
Supplement: Supplementary file 1 — Additional file 1: Text S1. Detailed description of materials, primers, antibodies and methods. [file 13104_2021_5785_MOESM1_ESM.docx]

**Materials and Methods**

Mouse strains and husbandry

*Dll1^AcGFPHAstopSF^* and *Dll1^SF^* mice were generated in this study (for details see later), ZP3::Cre [1] and FLPe deleter mice [2] were described previously. Mice were kept at the central animal facility of MHH under a 10/14h dark/light cycle at 21±2°C and 55±10% humidity in type2 L cages with wood bedding, without environmental enrichment. Food and water were provided ad libitum. Mice were maintained and analysed on a mixed (129Sv/ImJ; CD1; C57BL/6) genetic background. Wild type mice and embryos were used as controls, single animals were the experimental unit, except for the mass spectrometry and immune precipitations, where wild type or homozygous mutant embryos were pooled. Investigators could not be blinded to the mouse genotype due to the externally visible tail phenotype in adults or irregular somites in early embryos, and there were no confounders. As this was an exploratory descriptive study no statistical analyses were performed, no randomization was used, no exclusion criteria were set and none of the analysed mice/embryos was excluded. The study did not have humane endpoints. The number of adult females used for embryo collection from het to het matings was based on pilot studies to evaluate the amount of material required for immuno precipitations or affinity purifications.

Cell culture

CHO cells were cultured in DMEM/F12 medium 1:1 (Gibco), supplemented with penicillin/streptomycin, glutamax (Gibco) and 10% fetal calf serum (Biochrom). ES cells were grown in DMEM medium, supplemented with penicillin/streptomycin, glutamax, sodium pyruvate, essential amino acids (Gibco) and 15% fetal calf serum (Biochrom), β-mercaptoethanol and leukemia inhibitory factor and cultured on a layer of fibroblast cells as described previously [3].

Collection of embryos

Embryos were collected from heterozygous matings. Females were sacrificed by cervical dislocation, a fast method to sacrifice mice approved by the German authorities.

Generation of Constructs

*Modification of the Dll1 coding sequence*

To modify the 3´region of the *Dll1* gene and to add C-terminal tags a gene fragment was synthesized (GENEWIZ, UK) that spanned intron 10 and exon 11 including a unique HpaI site within intron 10 and a unique BsmI site in the 3´UTR in exon 11. 3’ to the last *Dll1* coding triplet (first triplet in exon 11) the gene synthesis contained sequences encoding monomeric AcGFP [4] fused in frame with an HA Tag followed by a stop codon, flanked by loxP sites. Downstream of the 3’ loxP site a SF tag and a stop codon [5] were included. The stop codon downstream of the HA tag prevented SF expression as long as AcGFPHA was present. Upon Cre-mediated recombination AcGFPHA was excised and SF expressed. 16 bp (AGGATATAGCCCCGAT; partial sequence of the guide RNA, see later) in the 3´UTR of *Dll1* were deleted to prevent cutting of the construct by the CRISPR/Cas system after electroporation of the targeting construct into ES cells. Downstream of exon 11 a *BamH*I and *Kpn*I site were included for insertion of the puromycin resistance cassette (Figure S1A Additional File 2).

*Constructs for analyses in cultured cells*

A *Dll1* „mini-gene“[6] consisting of exons1-10 as cDNA followed by intron 10 and exon 11 was subcloned as an EcoRI/NotI fragment in pGEM Teasy. The fragment obtained by gene synthesis was cloned as a HpaI/BsmI fragment into this vector resulting in the *Dll1* mini-gene with the modified 3´ end. This modified mini-gene was cloned as an EcoI/EcoRI 4,8kb fragment into pTracer CMV2, resulting in plasmid pCMV2DLL1AcGFPHAstopSF.

Cre-mediated recombination in SW106 Cre-expressing bacteria replaced AcGFPHA by SF in frame resulting in construct pCMV2DLL1SF (Figure 1A). Both constructs were stably integrated in CHO cells to express DLL1AcGFPHA and DLL1SF, respectively.

*Construct for targeting the Dll1 locus*

A 3’ portion of the *Dll1* mini-gene [6] from an NdeI site in exon9 to the EcoRI site downstream of exon11 was subcloned into pGEM Teasy. The fragment generated by gene synthesis was cloned as a *Hpa*I/*Bsm*I fragment into this vector and a FRT flanked PGK-puromycin-bGHpA resistance cassette was added as *BamH*I/*Kpn*I fragment (Figure S1A Additional File 2).

*CRISPR/Cas9 vector*

To improve the targeting frequency a double strand break was induced in exon 11 by CRISPR/Cas. The guide RNA was designed with the help of <http://crispr.edu/guides>http://crispr.edu/guides, and localized to the 3’ UTR of the *Dll1* gene. Guide RNA oligos (CACCGATTCATCGGGGCTATATCCT and AAACAGGATATAGCCCCGATGAATC) were annealed and cloned into the Addgene pX330 vector. Insertion of the annealed oligos was analysed by PCR using a forward primer in the Guide sequence and a reverse primer in the vector GGGGGAGATGGGGAGAGTGAAGC with the conditions 94°C 3min, 20x (94°C 30sec, 54°C 30sec, 72°C 45sec) amplifying a 500bp fragment verified by sequencing.

*Generation of CHO cell lines for in vitro analyses*

Expression plasmids pCMV2DLL1AcGFPHAstopSF or pCMV2DLL1SF were linearised by *Pvu*I, introduced into CHO cells using Perfectin according to the manufacturer’s instructions (Genlantis) and stable clones were established by Zeocin selection (250µg/ml). Expressing clones were identified by Western blot analysis using anti-DLL1 monoclonal antibody 1F9 (dilution 1:100 [7]) and the secondary antibody anti-rat-POD (1:10000 Amersham NA 935V).

*Cell surface biotinylation*

For each construct CHO cells stably expressing DLL1 variants were analyzed. 2,5 x 10^6^  cells of each cell line were seeded on 8 60mm dishes and treated after 24 hours at about 90% confluence. Cells were placed on ice and washed twice with ice-cold PBS C/M (1mM MgCl2; 0,1mM CaCl2), then incubated with 1,5 ml Sulfo-NHS-LC (0,25mg/ml in 1x PBS C/M, Thermo Scientific EZ-Link Sulfo NHS-LC Biotin #21335) for 40 min. After two consecutive washes with ice-cold PBS C/M the Biotin reaction was quenched by adding 100mM Glycin in DMEM and incubation for 30 min. Cells were washed with ice-cold PBS C/M and then lysed with 500 µl lysis buffer (50mM Tris/HCL pH7,6; 150mMNaCl, 1mM EDTA pH 8,0; 1% TritonX-100; 0,25% DOC, 0,1% SDS; Protease Inhibitor Complete Roche), harvested, sonified and centrifuged. To 50 microliter of the supernatant 50 microliter of 2xSB was added as an input. The remaining supernatant was incubated with 30 microliter NeutrAvidin beads (Thermo Scientific # 29200) overnight. Beads were washed 3x with lysis buffer, and 40 µl of 2xSB was added. Proteins were separated by SDS PAGE and transferred to membranes (Immobilon-P from Millipore). Membranes were cut between the 75 and 50 kDa size markers and analysed by Western blots, using antibody 1F9 (top part) for detection of DLL1, and anti β-Tubulin I antibody (Sigma T7816, 1:10^7^; secondary antibody: anti-mouse POD (Amersham NA 931V, 1:10^4^)) to detect accidental labelling of intracellular proteins with biotin in the IPs (bottom part). Eight samples per cell line were quantified relating the input band to the IP signal using Fiji.

*Gene targeting, screening and validation of ES cell clones*

75 µg of the targeting construct DNA linearized with *Sca*I and 75 µg of circular pX330 plasmid containing the guide RNA were introduced into 129/cast ES cells [3] via electroporation using GenepulserII and Capacitance Extender Plus (Biorad) set to 240V and 500 microF.. 48 hours after electroporation Puromycin was added at 1µg/ml medium. After 10 days 536 clones were picked onto 96 well plates. Upon confluence the 96 well plates were duplicated. Cells on one plate were lysed with 1x PCR buffer (500mM KCl,100mM Tris/HCl pH8.8, 0,1% Triton X-100) + Proteinase K (100µg/ml) overnight, followed by an incubation at 98°C for 3 min to inactivate the enzyme. Genomic DNA was analysed by PCR 94°C 3Min, 50x (94°C 30sec, 55°C 30sec, 72°C 1 min) for correct integration of at the 3´and 5´end. The 3´ region was analysed with primers puro F1 GGC TGG ACG TAA ACT CCT CTT CAG and Dll1 3´ R1 GTC ACT CTA ATG GAG ACA ACCTGC TAT G amplifying a 942 bp fragment. Positive clones were analysed with a second primer pair puro F2 AAG CGC ATG CTC CAG ACT GC and Dll1 3´ TTA GGA GAA GTC ACA AGG TCT CCG A resulting in a 1090bp fragment. Clones with correct 3’ integration were analysed by PCR for correct integration 5’ with primers Dll1 5´F1 GGC CCA TGG TGG TGG ACC and AcGFP R1 ATGGGCACGATGCCGGTG yielding a 670bp fragment and with primers Dll1 5´ F2 CTACATGTGTGAGTGCGCCCAG and AcGFP R2 GCTCACGCTGAACTTGTGGC resulting in a 790 bp fragment. 71 3’ and 5’ PCR positive clones were further analysed by Southern blot hybridisations for single integration events using a GFP probe that was obtained by PCR using primers F1 ATGAGCAAGGGCGCCGAGC and R1 CTTGTACAGCTCATCCATGCCGTGG with the conditions 94°C 3min, 30x (94°C 30sec, 63°C 30sec, 72°C 45sec) amplifying a 714bp fragment. Eight clones showing a single integration event were further analyzed by Southern blot hybridisations with DNA probes flanking the targeting vector 5’ and 3’. DNA fragments used as probes were obtained by PCR using 129/cast wt ES cell genomic DNA as template and conditions 94°C 3min, 30x (94°C 30sec, 56°C 30sec, 72°C 1min 30sec). The 5´probe was amplified with primers CAAGTCAATTCCGTGAGGATTCAGG and CTCTCACTGAGGTCCACCACCATG, resulting in a 1295 bp fragment, the 3´probe with primers CAGCTGTGAATCCAGGACTCTACTAG and CATCAGCTCACAGCCGGAAGTA resulting in a 1120 bp fragment. A 599bp probe from the puromycin resistance gene obtained by PCR using primers F1 ATGACCGAGTACAAGCCCACGGT and R1 CAGGCACCGGGCTTGCG with the conditions 94°C 3min, 30x (94°C 30sec, 57°C 30sec, 72°C 40sec) was used to confirm single integration events determined with the GFP probe. All amplified fragments used for probes were subcloned into pCRII Topo and verified by sequencing.

*Southern blot hybridisation*

Genomic ES cell DNA was digested with *BamH*I and separated by 0,6% agarose gel electrophoresis using the Gene Ruler 1kb Plus DNA Ladder from Thermo Scientific as a marker. Agarose gels were depurinated (0.2 N HCl; 10Min ), denatured (0,5N NaOH,1,5M NaCl 30Min), neutralized (1MTris/HCl pH8,1,5M NaCl, 30 Min) and blotted onto nylon membranes (Hybond-N+ Amersham) with 20xSSC overnight. Membranes were washed in 6x SSC and DNA cross-linked by UV. Blots were hybridised in hybridisation buffer (5xSSPE pH7.4, 5x Denhardt´s Solution, 100µg/ml sheared DNA, 0,5% SDS) overnight at 68°C. Probes were labelled with P^32^ dCTP with a Prime-It II random labelling Kit from Agilent according to the manufacturer´s instruction and purified with an illustra Quant G50 column from GE Healthcare. Hybridised blots were washed once in 2xSSC,0,1%SDS and once in 0,2xSSC,0,1%SDS and exposed for 2-4 days to Fujifilm BAS-IP MS 2025 phosphorescent imaging plates and scanned in a Fujifilm FLA7000 Laser scanner. Four ES cell clones were identified as correctly targeted (Figure S1B Additional File 2).

*Generation of mice carrying the modified Dll1 gene, removal of the puromycin-resistance cassette and replacement of AcGFPHA by Strep-Flag*

ES cells from two Southern blot positive clones were injected into d 2.5 embryos harvested from 20 females. Injected embryos were transferred by microsurgery into 7 pseudopregnant females that were anaesthetised by intraperitoneal injection of 100µl/10g body weight of a solution containing 8mg/ml ketamine, 0.2mg/ml xylazine, and 0.07mg/ml midazolam. In addition, 100µl/10g body weight of an atropine solution (5µg/ml) were injected subcutaneously to stabilise heartbeat. For analgesia 100µl/10g bodyweight of a meloxicam solution (0.1mg/ml) were administered subcutaneously.

1 clone transmitted the modified *Dll1* gene through the germ line. Presence of the transgene was detected by PCR using primers F1 ACCACTACCAGCAGAATACCCCCAT and R1 CTCGACTAGAGCTTGCGGAACCC with the conditions 94°C 3min, 50x (94°C 30sec, 63°C 30sec, 72°C 30sec) resulting in a 455bp fragment. The selection cassette was removed by crossing the *Dll1^AcGFPHAstopSF-puro^* mice to FLP-deleter mice resulting in the *Dll1****^Ac^****^GFPHAstopSF^* allele. Deletion of Puro was verified by PCR with primers F1 ACCACTACCAGCAGAATACCCCCAT and R1 CACACGTTTTCTTTTTCCCGTCTAGG with the conditions 94°C 3min, 50x (94°C 30sec, 60°C 30sec, 72°C 60sec) resulting in a 869 bp fragment. The AcGFPHA tag was excised by crossing *Dll1^AcGFPHAstopSF^* mice to ZP3:Cre mice resulting in the *Dll1^SF^* allele. Deletion of AcGFPHA was confirmed by PCR using primers TTATCTTGGAGCCACCCTCAGTTC and CACACGTTTTCTTTTTCCCGTCTAGG with the conditions 94°C 3min, 50x (94°C 30sec, 56°C 30sec, 72°C 45sec) resulting in a 580 bp PCR fragment. Both *Dll1* reporter mouse lines, *Dll1^AcGFPHAstopSF^* and *Dll1^SF^*, were kept on a hybrid background and bred to homozygosity, which was confirmed by the absence of a 498 bp PCR fragment that was obtained with wild type DNA with primers F1 GGGAGTGATGTGGAGAGGGTCCA and R1 CACACGTTTTCTTTTTCCCGTCTAGG with the conditions 94°C 3min, 40x (94°C 30sec, 56°C 30sec, 72°C 45sec). Subsequently, homozygous transgenic mice or embryos were typed phenotypically due to shortened and kinked tails or irregular somites (Figure 2B,E).

*Skeletal preparations*

d15.5 Mouse embryos (collected from 10 females of the different genotypes) were fixed with 100% Ethanol overnight. Cartilage was stained with Alcian blue solution (25ml 0,3% Alcian blue solution in 70% Ethanol, 100 ml acetic acid and 375 ml 100% ethanol) for 2 days at room temperature. Embryos were washed in 100% ethanol for 3 days followed by staining of the bones with Alizarin red solution (25 ml of 0,1% Alizarin Red S in 50% Ethanol, 125ml 2%KOH and 350ml H_2_O) over night. Embryos were treated with 1% KOH to clear soft tissues. Skeletons were documented with a Leica M420 microscope with Apozoom 1:6 and the software Photograb-300Z version 2.0.

*Confocal imaging*

Images were acquired at the MHH Imaging Core Facility using a Leica SP8 confocal laser microscope with a 10x or 20x air or immersion objective for whole-mount knock-in embryos and CHO cells, respectively. Embryos (collected from 1 wt and 3 *Dll1^AcGFPHAstopSF^* females) were mounted between two cover slips, CHO cells were plated on a glass-bottom dish (Mattek Model: P35G-0-10-C) and optical sections of 1 µm thickness were taken using the Las X Software (Leica) and processed with Adobe Photoshop CS5.

*Immunoprecipitation (IP)*

Wild type CHO cells and CHO cells expressing DLL1AcGFPHA or DLL1SF were grown to confluence in a 90 mm dish. For each IP one 90 mm dish was lysed with 1,5 ml lysisbuffer (50mM Tris/HCl pH7.5, 150mM NaCl, 1mM EDTA pH8.0,1%NP40,1%Triton X-100). Lysates were sonified and centrifuged for 30 minutes, at 13000rpm, 4°C. Supernatants were incubated overnight with anti-GFP (Roche 11 814 460 001‚ 5µl) or anti-HA (Roche 11 867 423 001, 10µl) or anti-Flag (Sigma F1804, 5µl) antibodies. 20 µl Sepharose G beads (GE Healthcare) washed with 20mM sodium phosphate buffer pH 7.0 were added to each probe and incubated overnight, then washed three times with washing buffer (50mM Tris/HCl pH7.5, 500mM NaCl, 5mM EDTA pH8.0, 05%NP40). Proteaseinhibitors from Serva (Protease Inhibitor-Mix M) or from Roche (cOmplete Tablets EDTA free) were added according to the manufacturers’ instructions. Beads were boiled in 2x SB and proteins analyzed by Western blots using anti-DLL1 antibody 1F9. The same procedure was applied for the immunoprecipitation of DLL1AcGFPHA or DLL1SF from lysates of E10,5 homozygous transgenic or wild type embryos using at least 10 embryos for each IP and genotype. A total of 50 females were used for embryo collection.

*Affinity purification of DLL1 complexes from DLL1-SF embryos and Mass Spectrometry*

Lysates of d10,5 embryos homozygous for the DLL1SF tag (total number of Tg/Tg embryos 455 obtained from 180 females) and wild type (total number of wt embryos 573 obtained from 230 females) as controls were used to purify tagged DLL1 complexes for mass spectrometry in six independent experiments. Embryos (exp 1-6: Tg/Tg 29e, 42e,71e,84e,94e,135e and wt 34e, 109e, 117e,96e,113e,104e) were lysed in (30mM Tris/HCl pH7.5,150mM NaCl, 0,5% Nonidet P40) 1-3 hours on ice. Embryo tissue was dissociated by pipetting up and down and lysates were centrifuged for 30 min at 13000rpm (Eppendorf) at 4°C. Supernatants were added to washed Anti-Flag M2 Affinity gel beads (Sigma) (1 µl per embryo), incubated overnight and put onto an Illustra Microspin Column (GE Healthcare), incubated overnight, then washed 3 times with about 500 µl washing buffer (30mM Tris/HCl pH7.5,150mM NaCl, 0,1% Nonidet P4) by flow through. Bound proteins were eluted with 200ng/µl Flag peptide (Sigma F3290) using about 2µl of Flag peptide solution/embryo. COmplete Tablets Roche plus Phosphatase Inhibitor Cocktail 2 (Sigma) plus Phosphatase Inhibitor Cocktail 3 (Sigma) 1:100 were used as protease inhibitors added to the lysis and washing buffer.

Eluates were subjected to methanol-chloroform precipitations followed by tryptic cleavage before they were analysed by LC-MS/MS as previously described [8]. Identification and quantification were performed with MaxQuant (https://maxquant.net/; version 1.6.1.09) [9,10] against the mouse subset of the Swissprot database (2019_08, #17,027 entries), statistical analysis was done with Perseus (version 1.6.2.3; https://maxquant.net/perseus/) [11]. The full data set has been included into the PRIDE database [12] and can be accessed under Accession number PXD024680.

**References**

1. de Vries WN, Binns LT, Fancher KS, Dean J, Moore R, Kemler R, et al. Expression of Cre recombinase in mouse oocytes: a means to study maternal effect genes. Genesis. 2000;26:110–2.

2. Rodríguez CI, Buchholz F, Galloway J, Sequerra R, Kasper J, Ayala R, et al. High-efficiency deleter mice show that FLPe is an alternative to Cre-loxP. Nat Genet. 2000;25:139–40.

3. Schuster-Gossler K, Cordes R, Müller J, Geffers I, Delany-Heiken P, Taft M, et al. Context-Dependent Sensitivity to Mutations Disrupting the Structural Integrity of Individual EGF Repeats in the Mouse Notch Ligand DLL1. Genetics. Genetics; 2016;202:1119–33.

4. Gurskaya NG, Fradkov AF, Pounkova NI, Staroverov DB, Bulina ME, Yanushevich YG, et al. A colourless green fluorescent protein homologue from the non-fluorescent hydromedusa Aequorea coerulescens and its fluorescent mutants. Biochem. J. Portland Press Limited; 2003;373:403–8.

5. Gloeckner CJ, Boldt K, Ueffing M. Strep/FLAG tandem affinity purification (SF-TAP) to study protein interactions. Curr Protoc Protein Sci. 2009;Chapter 19:Unit19.20.

6. Schuster-Gossler K, Cordes R, Gossler A. Premature myogenic differentiation and depletion of progenitor cells cause severe muscle hypotrophy in Delta1 mutants. Proc. Natl. Acad. Sci. U.S.A. 2007;104:537–42.

7. Geffers I, Serth K, Chapman G, Jaekel R, Schuster-Gossler K, Cordes R, et al. Divergent functions and distinct localization of the Notch ligands DLL1 and DLL3 in vivo. The Journal of Cell Biology. 2007;178:465–76.

8. Beyer T, Bolz S, Junger K, Horn N, Moniruzzaman M, Wissinger Y, et al. CRISPR/Cas9-mediated Genomic Editing of Cluap1/IFT38 Reveals a New Role in Actin Arrangement. Mol Cell Proteomics. American Society for Biochemistry and Molecular Biology; 2018;17:1285–94.

9. Cox J, Matic I, Hilger M, Nagaraj N, Selbach M, Olsen JV, et al. A practical guide to the MaxQuant computational platform for SILAC-based quantitative proteomics. Nature Protocols. 2009;4:698–705.

10. Cox J, Mann M. MaxQuant enables high peptide identification rates, individualized p.p.b.-range mass accuracies and proteome-wide protein quantification. Nat Biotechnol. Nature Publishing Group; 2008;26:1367–72.

11. Tyanova S, Temu T, Sinitcyn P, Carlson A, Hein MY, Geiger T, et al. The Perseus computational platform for comprehensive analysis of (prote)omics data. Nat Meth. 2016;13:731–40.

12. Vizcaíno JA, Csordas A, del-Toro N, Dianes JA, Griss J, Lavidas I, et al. 2016 update of the PRIDE database and its related tools. Nuc Acids Res. 2016;44:D447–56.
