# Supplementary figures and images for: Activity of the mouse Notch ligand DLL1 is sensitive to C-terminal tagging in vivo
Source: BMC Res Notes. 2021 Sep 28;14:383. doi: 10.1186/s13104-021-05785-4 (PMC8477538; doi:10.1186/s13104-021-05785-4)

A

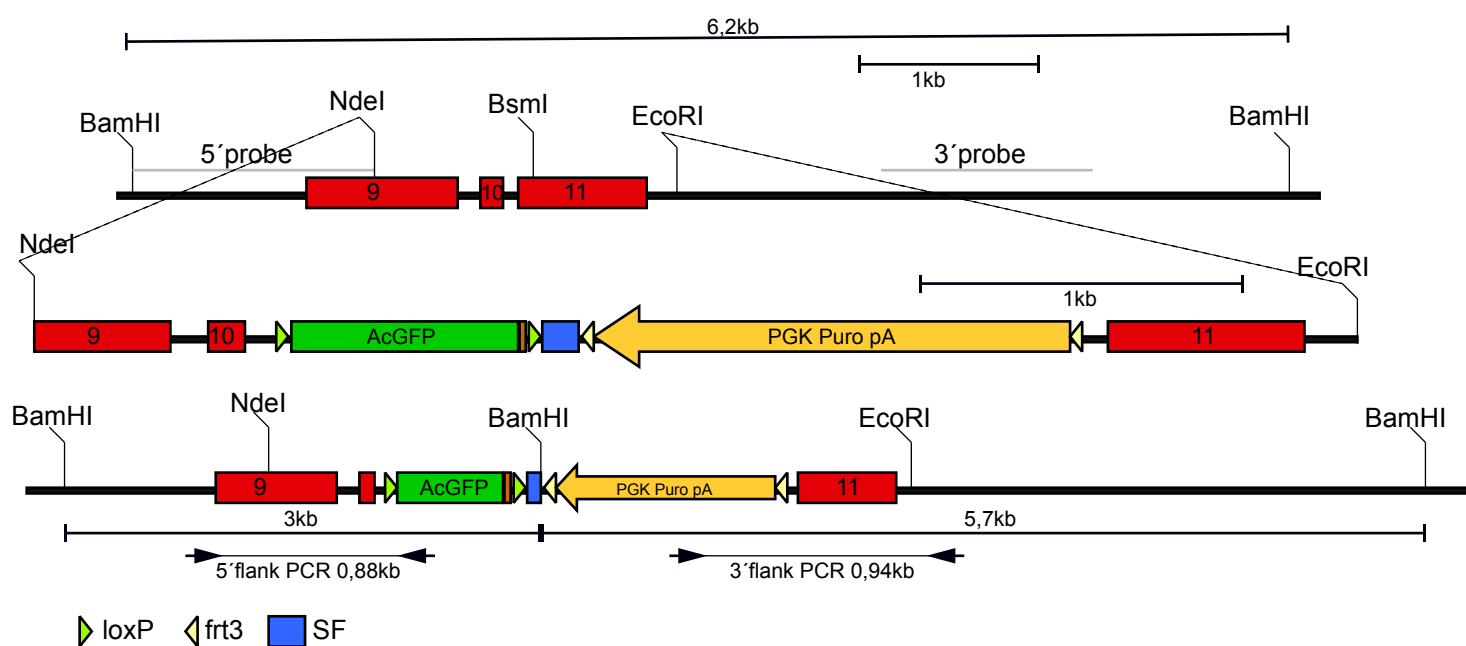

B

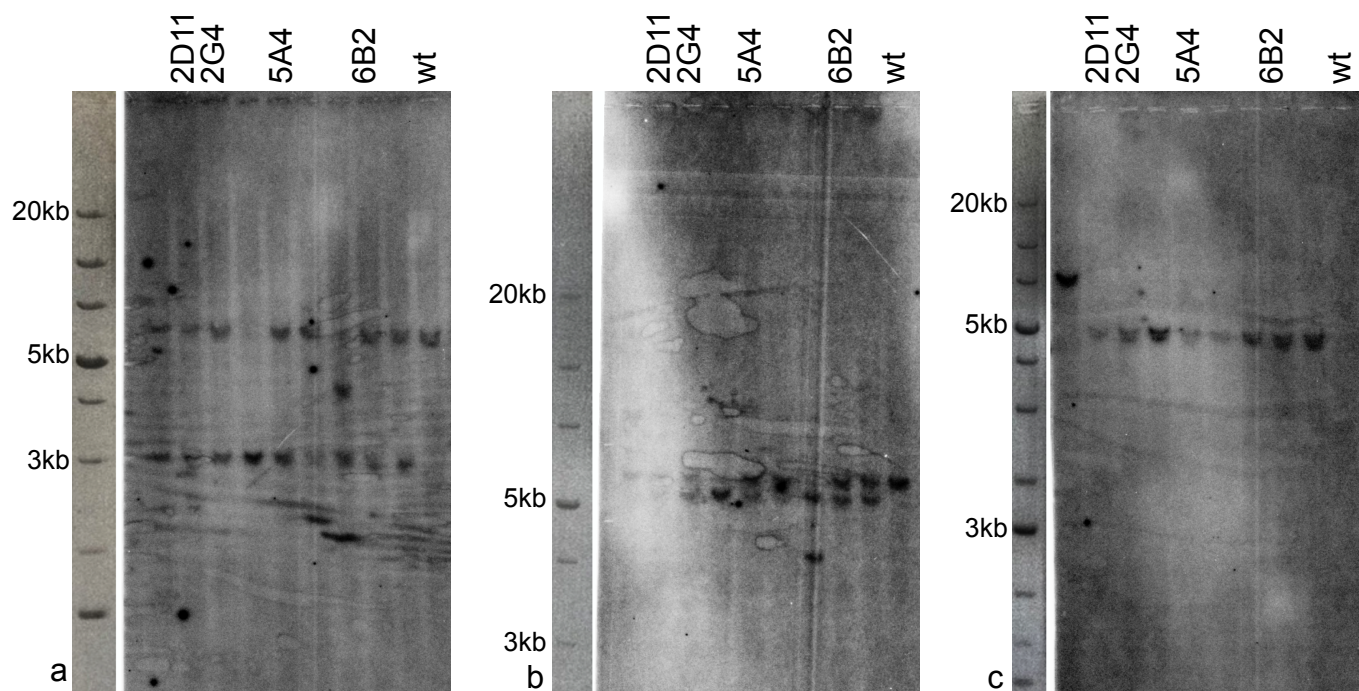

Supplement: Supplementary file 2 — Additional file 2: Figure S1. Targeting of Dll1. (A) Targeting scheme (for details see Material and Methods). (B) Southern blots of BamHI-digested genomic ES cell DNA with radioactively labelled probes from the 5’ flank (a), the 3’flank (b), and puro (c). Left lanes at each panel show ethidium bromide-stained lanes from agarose gels. Correctly targeted clones are indicated at the top. wt = wild type ES DNA. [file 13104_2021_5785_MOESM2_ESM.pdf]

# A DLL1AcGFPHA lysates

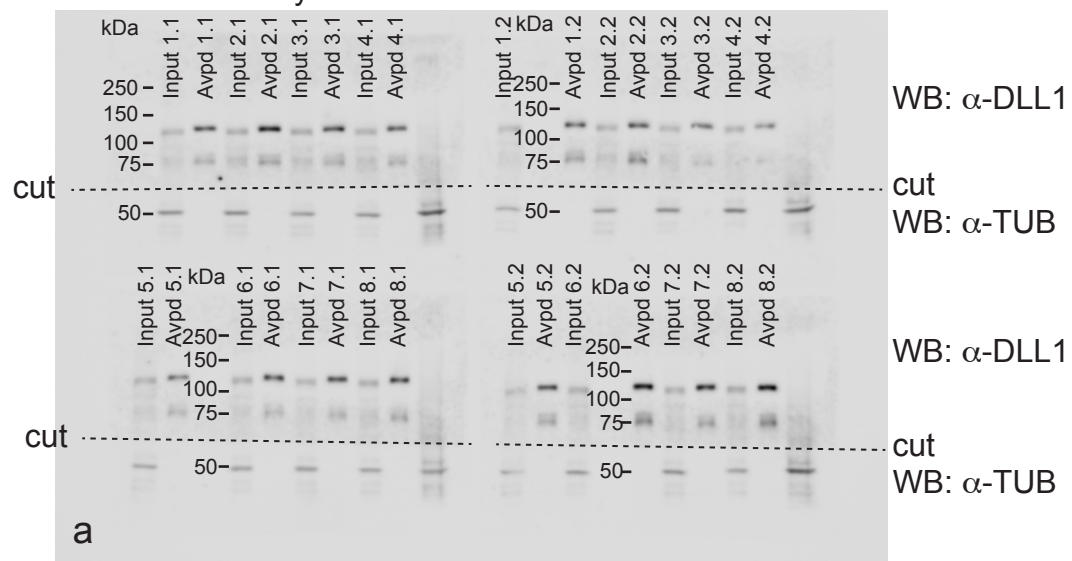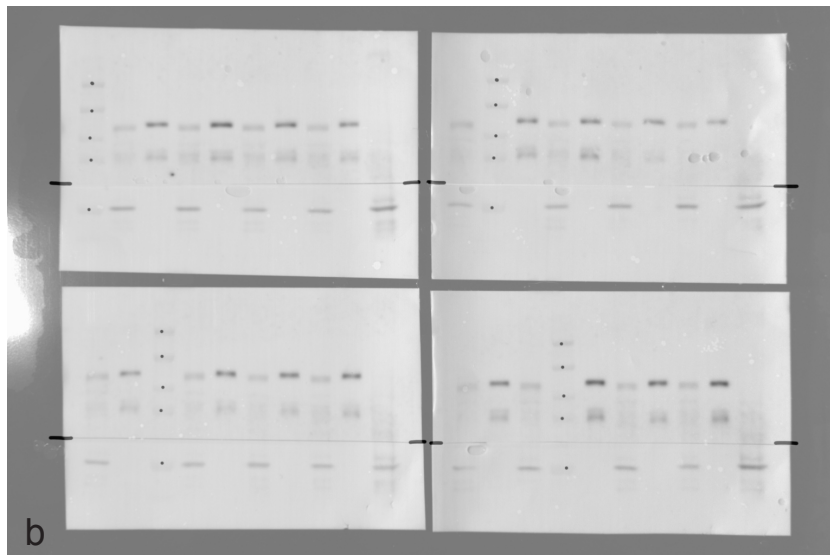

## B DLL1SF lysates

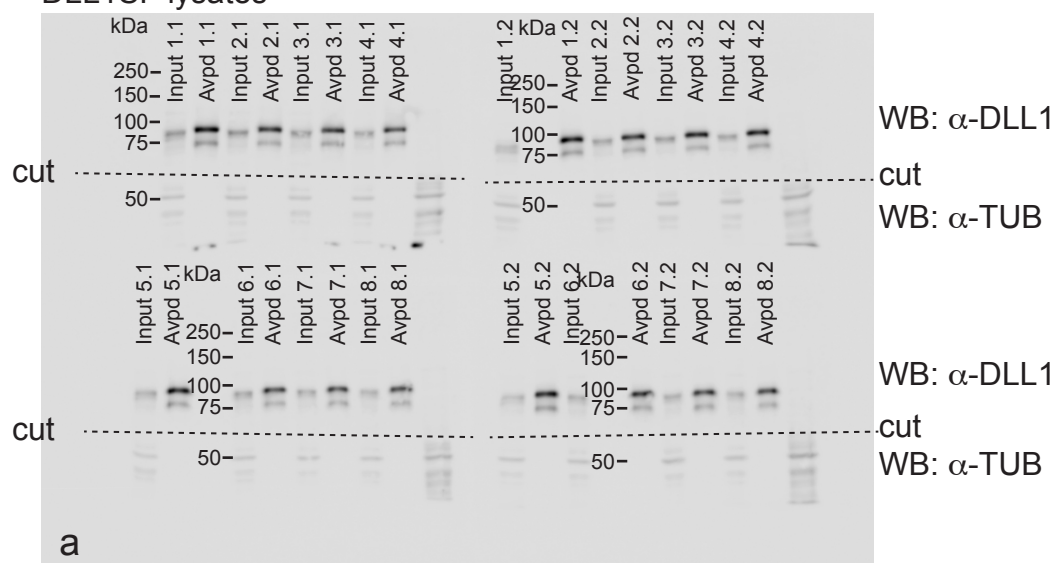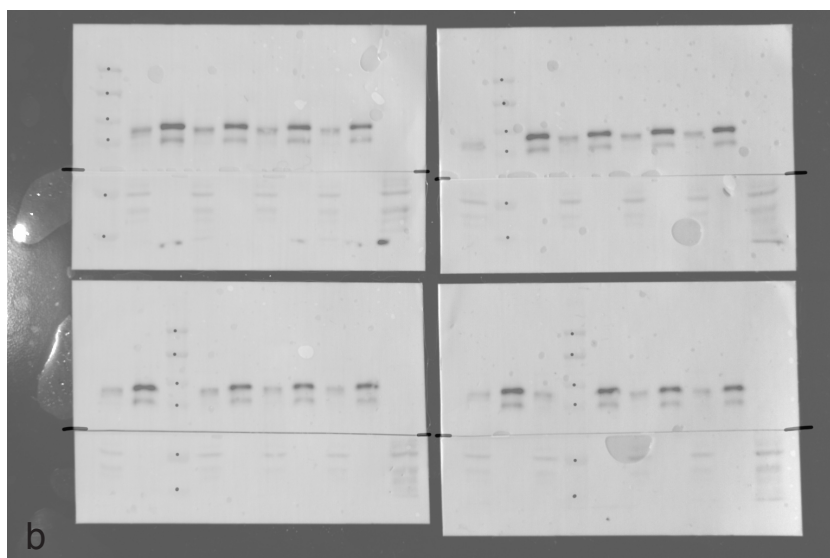

C

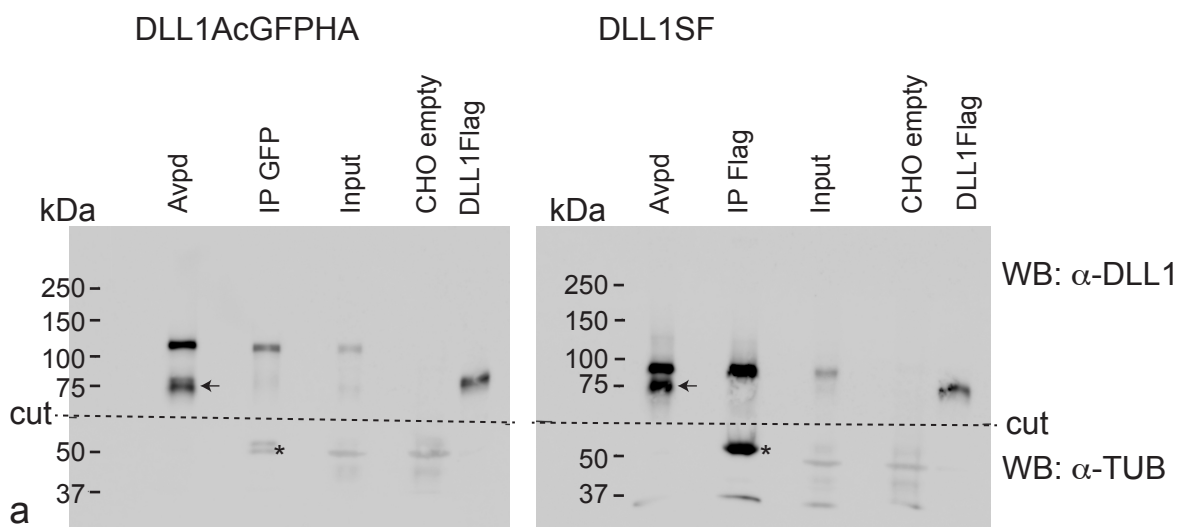

b

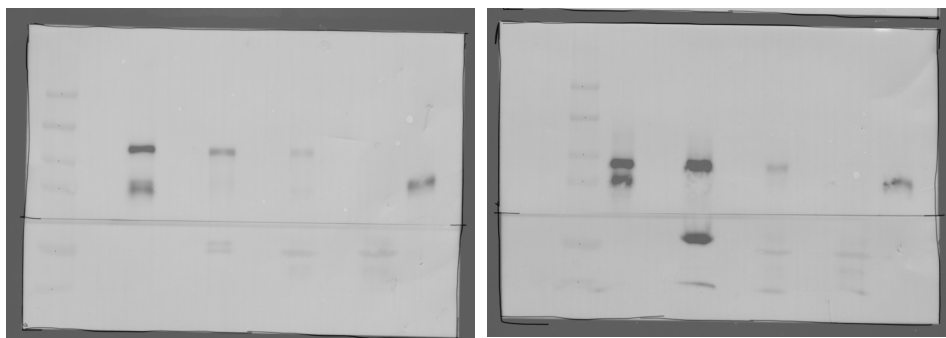

Supplement: Supplementary file 3 — Additional file 3: Figure S2. Surface biotinylation of tagged DLL1 proteins. (A) Western blots of cell lysates (input) and biotinylated proteins purified by Avidin beads (Avpd) from CHO cells expressing DLL1AcGFPHA. (a) Photograph of bound antibodies detected by chemoluminescence, (b) overlay of bright field and chemoluminescence photographs of Western blot membranes. Two aliquots from each of the 8 samples analysed per cell line (x.1 and x.2) were quantified relating the input to the Avpd band. Dotted lines indicate where membranes were cut. Primary antibodies used are indicated to the right. (B) Western blots of cell lysates (input) and biotinylated proteins purified by Avidin beads (Avpd) from CHO cells expressing DLL1SF. (a) Photograph of bound antibodies detected by chemoluminescence, (b) overlay of bright field and chemoluminescence photographs of Western blot membranes. Two aliquots from each of the 8 samples analyzed per cell line (x.1 and x.2) were quantified relating the input to the Avpd band. Dotted lines indicate where membranes were cut. Primary antibodies used are indicated to the right. (C) Western blots of cell lysates (input) and biotinylated proteins purified by Avidin beads (Avpd) or immunoprecipitated with anti-GFP (IP GFP) or anti-Flag (IP Flag) antibodies from CHO cells expressing DLL1AcGFPHA (left) or DLL1SF (right). (a) Photograph of bound antibodies detected by chemoluminescence, (b) overlay of bright field and chemoluminescence photographs of Western blot membranes. Dotted lines indicate where membranes were cut. Primary antibodies used are indicated to the right. DLL1Flag: lysate of CHO cells expressing flag-tagged DLL1 serving as positive control. Arrows point to biotinylated DLL1 purified by Avidin beads that is not immunoprecipitated by anti-GFP or anti-Flag antibodies. Asterisks indicate Ig heavy chains of primary antibodies used for immunoprecipitations detected by the secondary antibodies. [file 13104_2021_5785_MOESM3_ESM.pdf]

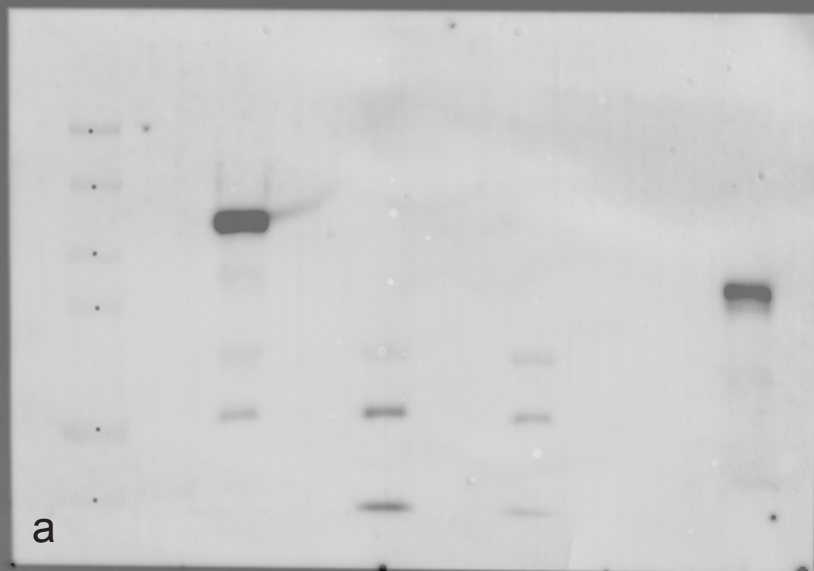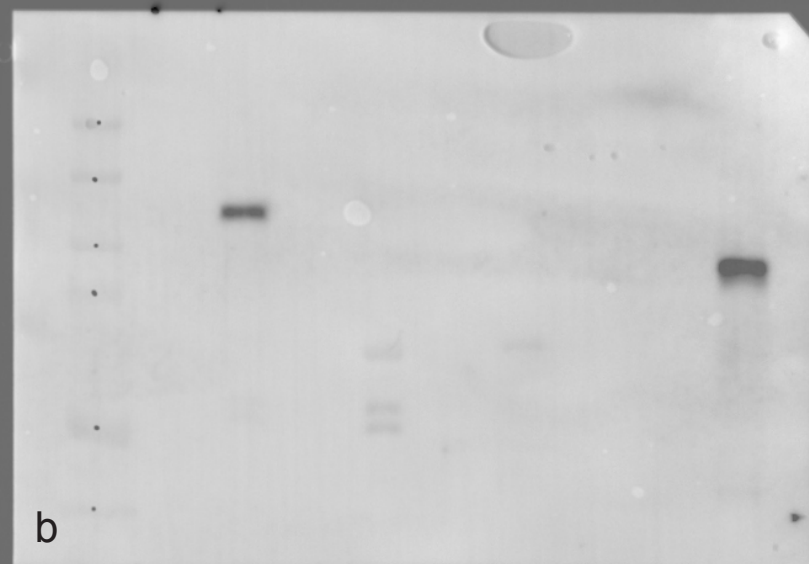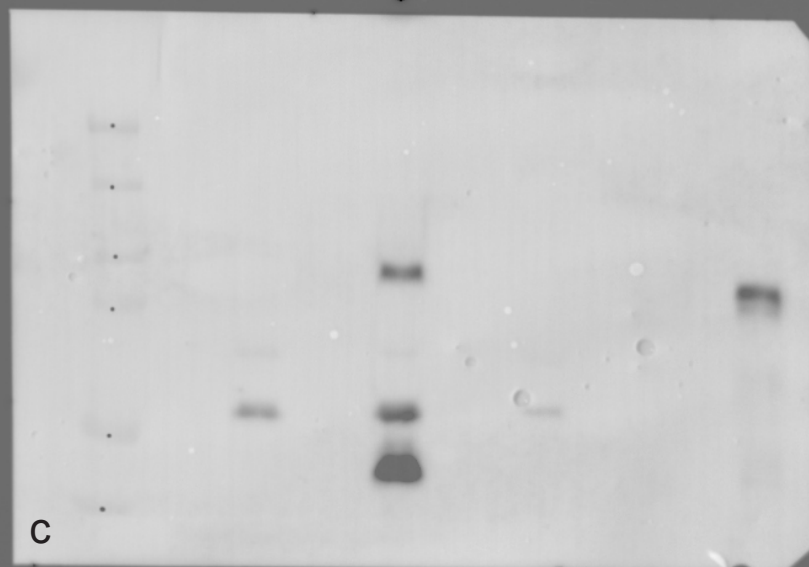

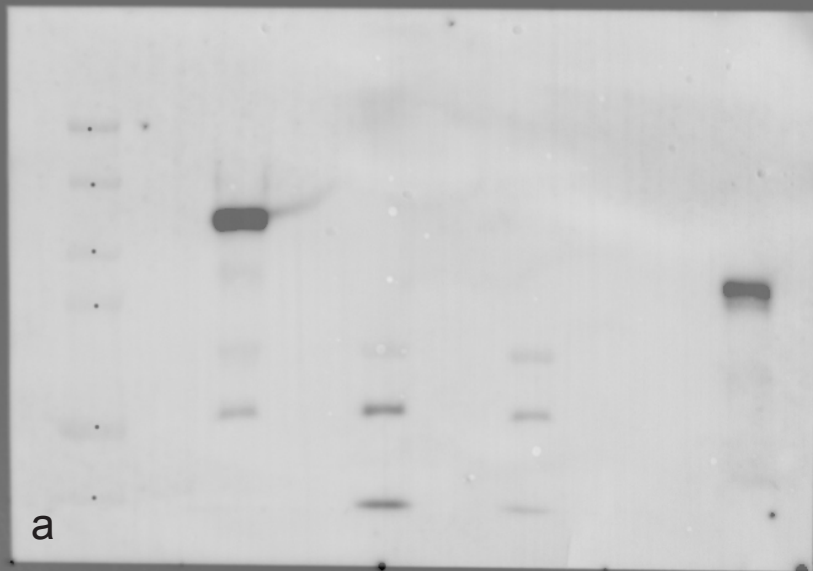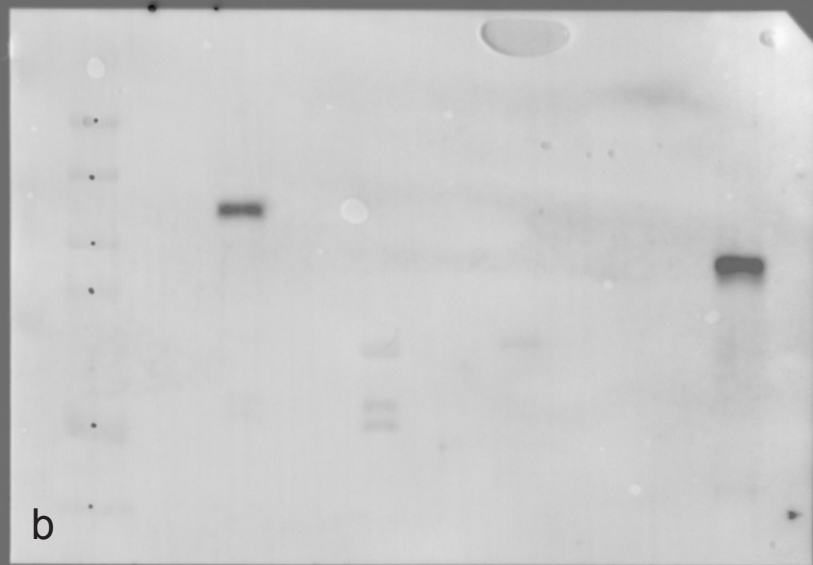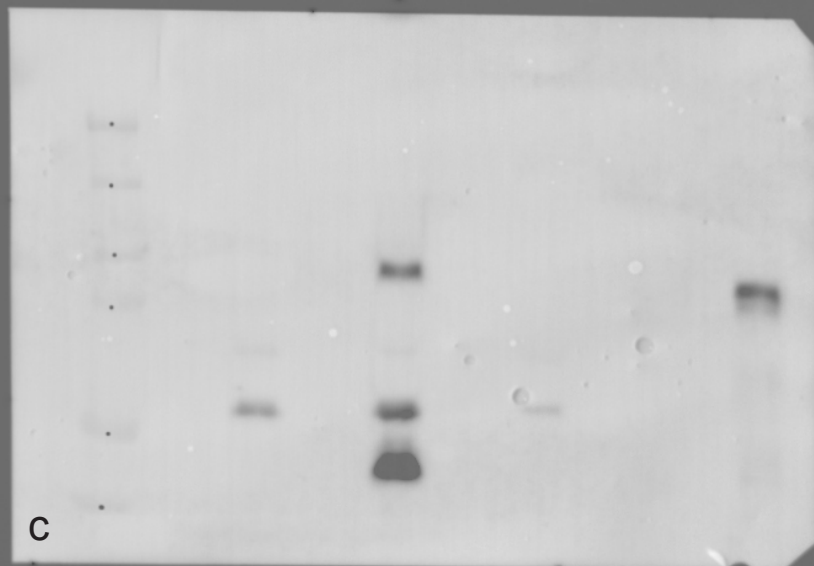

Supplement: Supplementary file 7 — Additional file 7: Figure S3. Overlay of bright field and chemoluminescence photographs of the Western blot membranes used for Fig. 1Ba-c. a-c correspond to a-c in Fig. 1B. [file 13104_2021_5785_MOESM7_ESM.pdf]

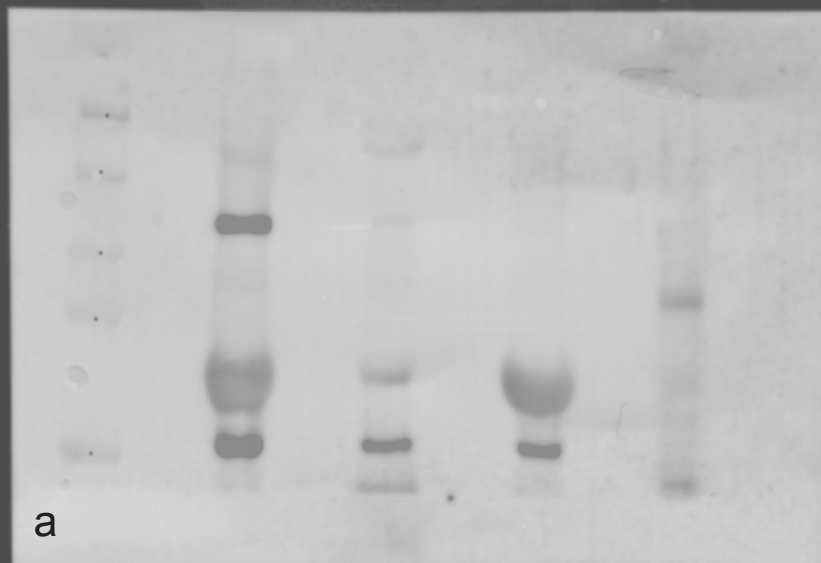

a

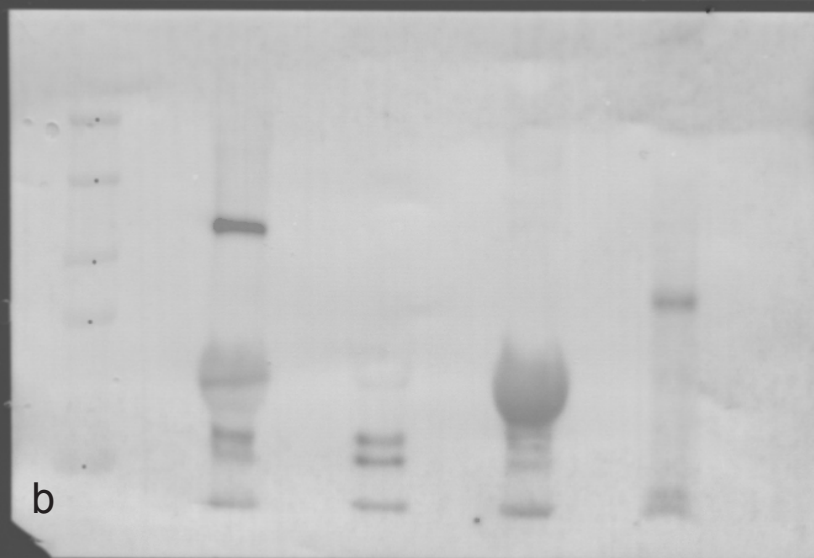

b

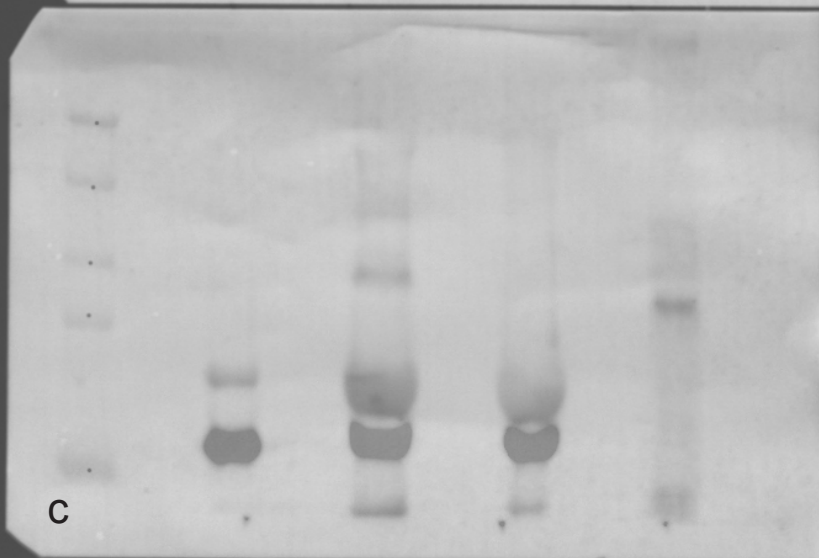

c

Supplement: Supplementary file 8 — Additional file 8: Figure S4. Overlay of bright field and chemoluminescence photographs of the Western blot membranes used for Fig. 2D a-c. a-c correspond to a-c in Fig. 2D. [file 13104_2021_5785_MOESM8_ESM.pdf]
